# Auditory brainstem responses are resistant to pharmacological modulation in Sprague Dawley wild-type and Neurexin1α knockout rats

**DOI:** 10.1186/s12868-024-00861-4

**Published:** 2024-03-15

**Authors:** Samuel Marashli, Philipp Janz, Roger L Redondo

**Affiliations:** 1 Roche Pharma Research and Early Development, Neuroscience and Rare Diseases, Roche Innovation Center Basel, F. Hoffmann-La Roche Ltd., Grenzacherstrasse 124, 4070 Basel, Switzerland; 2 Neuroscience Center Zurich, University of Zurich and ETH Zurich, Zurich, Switzerland

**Keywords:** Auditory brainstem responses, Pharmacological modulations, Neurexins, Non-invasive brain technology, Neurophysiology

## Abstract

Sensory processing in the auditory brainstem can be studied with auditory brainstem responses (ABRs) across species. There is, however, a limited understanding of ABRs as tools to assess the effect of pharmacological interventions. Therefore, we set out to understand how pharmacological agents that target key transmitter systems of the auditory brainstem circuitry affect ABRs in rats. Given previous studies, demonstrating that Nrxn1α KO Sprague Dawley rats show substantial auditory processing deficits and altered sensitivity to GABAergic modulators, we used both Nrxn1α KO and wild-type littermates in our study. First, we probed how different commonly used anesthetics (isoflurane, ketamine/xylazine, medetomidine) affect ABRs. In the next step, we assessed the effects of different pharmacological compounds (diazepam, gaboxadol, retigabine, nicotine, baclofen, and bitopertin) either under isoflurane or medetomidine anesthesia. We found that under our experimental conditions, ABRs are largely unaffected by diverse pharmacological modulation. Significant modulation was observed with (i) nicotine, affecting the late ABRs components at 90 dB stimulus intensity under isoflurane anesthesia in both genotypes and (ii) retigabine, showing a slight decrease in late ABRs deflections at 80 dB stimulus intensity, mainly in isoflurane anesthetized Nrxn1α KO rats. Our study suggests that ABRs in anesthetized rats are resistant to a wide range of pharmacological modulators, which has important implications for the applicability of ABRs to study auditory brainstem physiology.

**Supplementary Information:**

The online version contains supplementary material available at 10.1186/s12868-024-00861-4.

## Introduction

Auditory brainstem responses (ABRs), also known as brainstem auditory evoked potentials, are electrical potentials commonly evoked by click sounds, which can be measured non-invasively and that speak to synaptic transmission within the auditory brainstem circuits. ABRs are widely used for assessing hearing thresholds [1], intraoperative neuromonitoring [2], screening for sensory abnormalities in neurodevelopmental disorders [3], or testing ototoxicity in drug development [4].

In both humans and rodents, ABRs consist of distinct deflections (also referred to as ‘waves’), that are generated by the activation of specific neuronal nuclei within the auditory pathway [5–7]. We can differentiate between four to five waves, with a temporal separation of about 0.8–1.0 ms each [8]. Wave I is generated by the distal part of the auditory nerve (AN). Wave II reflects the projection of the cochlear nucleus (CN); Wave III is generated by the superior olivary complex (SOC), wave IV by the lateral lemniscus and inferior colliculus (IC), and lastly wave V reflects signal transmission from the thalamus to the auditory cortex (AC) [9–11].

The neurotransmitter systems in the auditory brainstem circuitry are mainly glutamatergic, GABAergic, glycinergic, and cholinergic [12–14]. The ventral part of the cochlear nucleus sends glutamatergic projections to the lateral superior olive (LSO), the medial superior olive, and the medial nucleus of the trapezoid body (MNTB), while the dorsal cochlear nucleus send glutamatergic projections to the IC. MNTB neurons make glycinergic inhibitory synapses with the LSO neurons. SOC neurons send glutamatergic projections to the lateral lemniscus and the IC, targeting the medial geniculate body in the thalamus, which sends glutamatergic projections again to the AC [12, 13]. The descending auditory projections start from the AC and terminate in subcortical auditory centers, such as the IC in the auditory brainstem [15, 16].

While ABRs have been used extensively to assess auditory brainstem physiology [17] and its abnormalities [18, 19], the capacity of ABRs to be modulated by pharmacological agents remains poorly understood. Therefore, we set out to test the effects of various pharmacological modulators on rodent ABRs. Here we used acute pharmacological treatments prior to the ABR measurements. We tested the effects of enhancing the GABAergic neurotransmission in the auditory brainstem via injecting diazepam (a γ2-containing GABA_A_ receptor enhancer), gaboxadol (a δ-containing GABA_A_ receptor agonist) or baclofen (a GABA_B_ receptor agonist). Both GABA_A_ and GABA_B_ receptors are widely expressed along the different nuclei in the auditory brainstem [20–24]. A previous study emphasized the role of baclofen and diazepam as potent modulators of both the excitability of neurons in the ascending auditory pathway and the processing of auditory information by IC neurons [25]. Moreover, we used bitopertin (a non-competitive selective inhibitor of glycine transporter 1 (GlyT-1) [26]) to investigate the role of increased glycinergic neurotransmission on ABRs. GlyT-1 is one of the two glycine transporters family, which work as an endogenous regulator of glycine, but also play a crucial role in maintaining glycine neurotransmission homeostasis and modulating glycine levels at N-methyl-D-aspartic acid (NMDA) sites [26]. GlyT-1 is widely expressed in neuronal and glial cells [27], among the different brain regions including the auditory brainstem [28]. We also used retigabine (a broad K_v_7 enhancer), which is well known to increase neuronal hyperpolarization [29] and thus may reduce synaptic outputs in the auditory brainstem by acting on the K_v_7.4 channels of the outer hair cells in the inner ear [30]. In addition, we used nicotine, a nicotinic acetylcholine receptor (nAChR) agonist, to inhibit excitatory output of the outer hair cells in the cochlea [31].

Initially, we tested these compounds under the application of isoflurane, a frequently used anesthesia method for rodent ABRs [1]. In a second step, we also tested a subset of compounds under medetomidine anesthesia that may better preserve the dynamics of neural circuits and therefore could reveal compound effects different from those under isoflurane. Furthermore, by using three well-regarded anesthesia methods (isoflurane, ketamine/xylazine, and medetomidine), we compared the ABRs between Nrxn1α KO rats and wildtype littermates under the most frequently used anesthetic conditions. In humans, a 2p16.3 (NRXN1) deletion is associated with intellectual disability, autism spectrum disorder, and schizophrenia [32]. Previously, we showed that auditory processing is substantially impaired in Nrxn1α KO rats, and that cortical auditory responses are impacted differently by GABAergic modulation compared to their wild-type littermates [33]. Therefore, the inclusion of Nrxn1α KO Sprague Dawley rats allowed us to test if functional alterations of auditory brainstem circuits could explain some of our previous results.

## Materials and methods

### Animals

Experiments were conducted on adult Nrxn1α KO rats and wild-type littermates (strain: Sprague Dawley (SD)-Nrxn1 < tm1sage > bred by Charles River, France. Only male rats were used. Rats were housed in groups of two, in a temperature-controlled room on a 12 h light/dark cycle with ad libitum food and water. Overall, four animal cohorts have been used, since is not feasible to run all tests in a single cohort given limitations from age-effects and animal welfare perspective, as Table 1 shows.

**Table 1:** Shows the different animal cohorts used according to each pharmacological treatment and anesthesia type in both wild-type (WT) and Nrxn1α KO Sprague Dawley rats (KO). N/A: pharmacological treatment is not applicable

Animal Cohort	Anesthesia type	Pharmacology	Animal numbers	Animal age
Group A	Isoflurane	Nicotine	WT (N = 14), KO (N = 14)	20 weeks old
		Bitopertin	WT (N = 14), KO (N = 11)	
		Baclofen	WT (N = 14), KO (N = 13)	
	Ketamine/xylazine	N/A	WT (N = 9), KO (N = 9)	25 weeks old
Group B	Medetomidine	Diazepam	WT (N = 12), KO (N = 12)	25 weeks old
		Bitopertin		
		Retigabine		
		N/A		27 weeks old
	Isoflurane	N/A		
Group C	Isoflurane	Diazepam	WT (N = 14), KO (N = 14)	20 weeks old
		Gaboxadol		
Group D	Isoflurane	Gaboxadol	WT (N = 18), KO (N = 16)	20 weeks old
		Retigabine		

All procedures were approved by the Federal Food Safety and Veterinary Office of Switzerland (Basel) and conducted in adherence to the Swiss federal ordinance on animal protection and welfare, as well as according to the rules of the Association for Assessment and Accreditation of Laboratory Animal Care International and the ARRIVE guidelines [34]

### Anesthesia

Isoflurane-based anesthesia started with inducing unconsciousness via isoflurane inhalation (Isofluran Baxter, Cat. no.: hdg9623, Baxter, GER), in a chamber filled with 5% isoflurane for 3 min and maintained throughout the ABR recordings at 2.5% isoflurane in medical air.

For medetomidine-based anesthesia, animals were first anesthetized via isoflurane inhalation (4% isoflurane for 4 min), and then injected with a bolus of medetomidine (0.1 mg/kg, s.c., Dorbene, Graeub, CH), followed by 1 min isoflurane inhalation at 4% to maintain anesthesia until the effect of medetomidine fully unfolded. Before starting the ABR measurements, isoflurane inhalation was stopped for 5 min to ensure isoflurane washout. At the end of the recording, Atipamezoli (0.1 mg/kg, s.c., Alzane, Graeub, CH) was injected to reverse the sedative and analgesic effects of medetomidine. Ketamine-based anesthesia was performed by i.p injection of a ketamine/xylazine mixture (80 mg/kg ketamine mixed with 5 mg/kg xylazine, i.p., Ketasol 100 with Xylasol, Graeub, CH). ABR measurements were started 10 min after injection.

### Pharmacology

Doses and pre-treatment times were chosen according to previously established pharmacokinetic/pharmacodynamics profiles [33, 35–39]. Treatment conditions were randomized using a Latin-based square design (also referred to as “William’s design”), in which each animal received every compound (or vehicle) in a randomized fashion. The randomization controls for putative day-to-day variability and allows within-subject comparison strengthening statistical power, in line with previous neuropharmacological studies [33]. No blinding was performed. The duration of the washout phase between dosing was at least 48 h. The control condition was represented by the administration of an equal volume of the vehicle solution (0.9% saline + 0.3% Tween20: Cat. no.: 11332465001, Sigma-Aldrich, GER). Animals were injected with diazepam (3 mg/kg, Roche Pharmaceuticals, CH), gaboxadol (10 mg/kg, Cat. no.: T101, Sigma-Aldrich, GER), retigabine (3 mg/kg, Roche Pharmaceuticals, CH), nicotine (5 mg/kg, ( −) nicotine hydrogen tartrate salt, Cat. no.: SML1236, Sigma–Aldrich, GER), baclofen (5 mg/kg, Cat. no.: B5399, Sigma–Aldrich, GER), bitopertin (10 mg/kg, Roche Pharmaceuticals, CH) or vehicle solution. Intraperitoneal injection was performed 15 min before starting the ABR measurements for all compounds, except for bitopertin, which reaches maximal exposures at around 60 min after application. Given the time necessary for the preparation (anesthesia, placing of the animal in to the recording device and positioning the electrodes) the actual ABR recordings happened about 30 min post-dosing (or at 75 min in case of bitopertin).

### Electrophysiological recording and acoustic stimulation

Prior to the ABR measurements, sound volume calibration was performed following the RZ6 Open Field Calibration Setup (Tucker-Davis Technologies, FL), including a signal conditioner and a 1/4-inch Prepolarized Free-field microphone (model nr. 480c02, ICP® SENSOR, PCB, NY, USA). The acoustic stimuli used in the ABRs assessment consisted of 512 click sounds, generated at 200 kHz sampling rate. Each click sound is a broadband mono-phasic square wave signal (0.1 ms). The click sounds were presented at a rate of 21 clicks/s, at different sound levels (90, 80, 70, 60, 50, 40, 30, 20, 10 dB SPL), starting with the highest stimulus intensities, in line with established protocols [33, 40]. The ABR measurements were conducted in a sound-attenuating and electrostatically grounded chamber. Body temperature of anesthetized animals (see above) was maintained at 37^◦^ C using a thermic heating pad (Kent Scientific Corporation, CN, USA). Click sounds were generated with a multi-field speaker (MF1, Tucker-Davis Technologies, FL, USA) connected to a RZ6-A-1 input/output processor (Tucker-Davis Technologies, FL, USA). The speaker was positioned 10 cm from the animal’s right ear. ABR signals were recorded with 13 mm subdermal needle electrodes (Cat. no.: NS-s83018-r9-10, Rochester, Coral Springs, FL, USA), with the signal electrodes placed on the vertex and reference and ground electrodes placed under the ipsi- and contralateral ear, respectively, connected to a RA4PA preamplifier/digitizer and RA4LI low impedance head stage (Tucker-Davis Technologies, FL, USA). Signals were acquired using the following settings: 12 kHz sampling rate, 5 kHz low pass, 100 Hz high pass, 50 Hz notch, using the BioSigRZ software (version 5.5, TDT, FL, USA).

### Euthanasia

At the end of the experimental producers, animals received terminal anesthesia; 150 mg/kg pentobarbital (Eskonarkon, Switzerland), i.p.,1:20 dilution with NaCl, followed by decapitation, after confirming a lack of reflexes by paw pinching.

### Data processing and analysis

Data analysis was performed as previously described [33]. In brief, in a pre-processing step ABR data were normalized to its pre-stimulus baseline. Resulting ABR waveforms were statistically tested for differences between conditions (see Statistical testing).

### Statistical testing

Statistical testing was performed with paired or unpaired cluster-based permutation tests (CBPT) depending on the condition, using custom *Python* scripts. In brief, first CBPT performs individual t-tests (two-tailed, significance level set to p < 0.05) for each data point. The resulting clusters are then tested for significance by comparing the summed t-values of the initial clusters with summed t-values of clusters obtained from permuted data (here, shuffling over the time domain) over many iterations (N = 1000 permutations, significance threshold: p < 0.05), thereby correcting for multiple comparisons. We visualize both cluster types (with permutation testing: black bars above graphs; and w/o permutation: grey bars, indicating statistical trends). Given that qualitatively no apparent outliers were present, no specific test was performed for outlier detection. No exclusion criteria were predetermined, and no animals were excluded from the statistical analysis. For one animal under one condition in the pharmacology study (nicotine, 5.0 mg/kg), missing vehicle data were input by averaging the respective data points of all other animals under this condition, to allow for paired analyses.

## Results

### Auditory brainstem responses are similar for adult Nrxn1α KO Sprague Dawley rats and wild-type littermates under different anesthetics

**Fig. 1:**
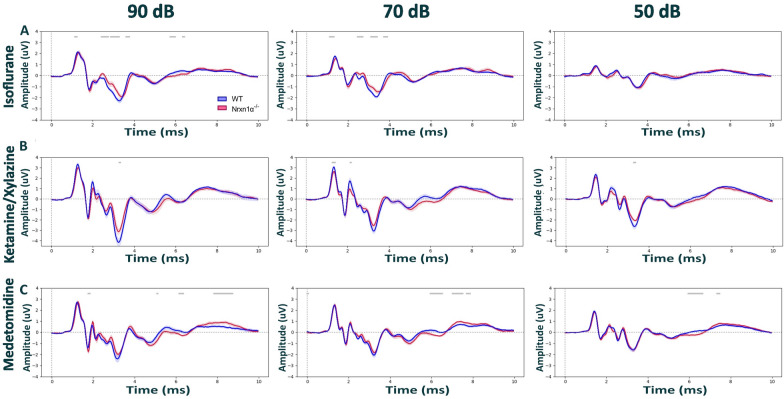
Comparison of auditory brainstem responses between Nrxn1α KO Sprague Dawley and wild-type littermates rats. ABR waveforms across different stimulus intensities (90, 70, 50 dB) under **A** isoflurane, **B** ketamine/xylazine and **C** medetomidine anesthesia. Recordings from the WT are in blue (N = 12) and Nrxn1α KO in red (N = 12). Data displayed as mean ± SEM, was tested with unpaired CBPT. No robust significant differences were found between genotypes across anesthesia methods. Grey bars above the graphs indicate clusters of significant differences before CBPT-based correction for multiple comparisons, i.e., indicating statistical trends

First, we asked whether Nrxn1α KO Sprague Dawley rats show alterations in their ABRs compared to wild-type littermates. To mitigate the risk that putative genotypic differences are missed due to the effects of anesthesia, we performed ABR recordings under three different types of anesthesia. We found that under all conditions, ABRs of Nrxn1α KO animals largely resembled those of their wild-type littermates (Fig. 1 and Additional file 1: Figs. S1 and S2). Except for statistically significant differences in the very late components of the ABRs elicited at 80 dB under medetomidine (Additional file 1: Fig. S2B; time window 5.4–6.5 ms, d = − 1.23, p = 0.028 and time window 7 – 8.5 ms, d = 1.12, p = 0.012).

### ABRs are largely resistant to pharmacological modulators under isoflurane anesthesia

**Fig. 2:**
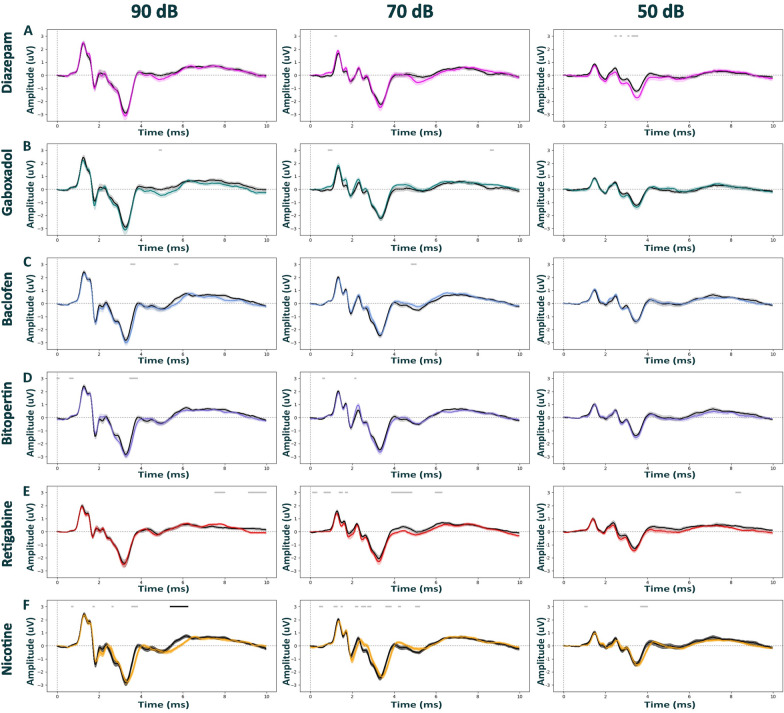
Auditory brainstem responses post pharmacological treatment in WT Sprague Dawley rats under isoflurane anesthesia. ABR waveforms across different stimulus intensities (90, 70, 50 dB) post intraperitoneal injection with diazepam (3 mg/kg) in magenta; (N = 14), gaboxadol (10 mg/kg) in teal; (N = 14), baclofen (5 mg/kg) in blue; (N = 14), bitopertin (10 mg/kg) in purple, retigabine (3 mg/kg) in red; (N = 18), nicotine (5 mg/kg) in yellow; (N = 14),; (N = 14), or vehicle solution in black (0.9% saline + 0.3% Tween). Within each experimental block, dosing was counterbalanced, and applied 15 min prior to the ABR recordings for all compounds, except for bitopertin (60 min pre-treatment time). The Black bars above the graphs indicate clusters of significant differences between conditions. The Gray bars indicate clusters that have not reached significance threshold post-permutations. Data displayed as mean ± SEM

**Fig. 3:**
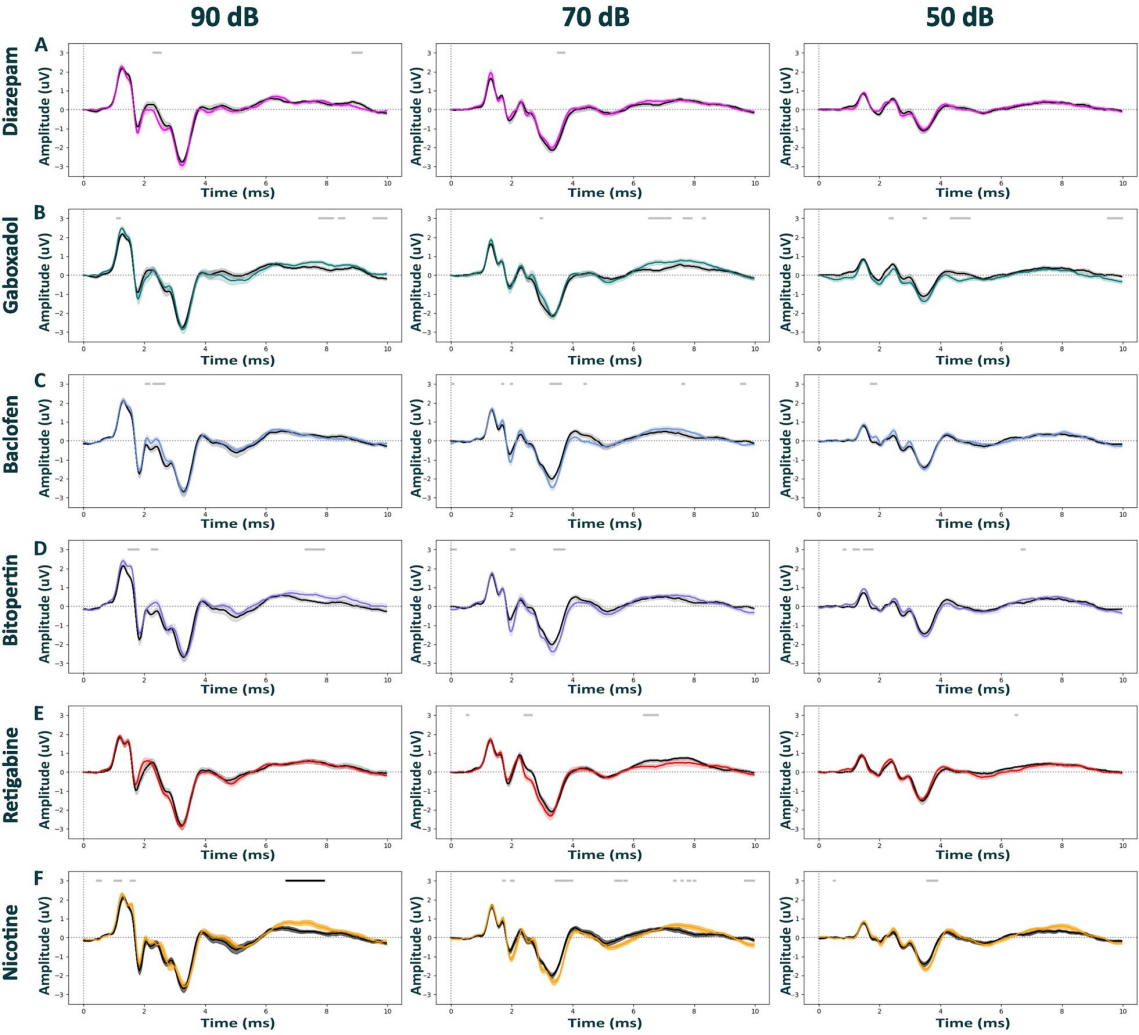
Auditory brainstem responses post pharmacological treatments in Nrxn1α Sprague Dawley rats under isoflurane anesthesia. ABR waveforms across different stimulus intensities (90, 70, 50 dB) post intraperitoneal injection with diazepam (3 mg/kg) in magenta; (N = 14), gaboxadol (10 mg/kg) in teal; (N = 14), baclofen (5 mg/kg) in blue; (N = 13), bitopertin (10 mg/kg) in purple; (N = 11), retigabine (3 mg/kg) in red; (N = 16), nicotine (5 mg/kg) in yellow; (N = 14), or vehicle solution in black (0.9% saline + 0.3% Tween). Within each experimental block, dosing was counterbalanced, and applied 15 min prior to the ABR recordings for all compounds, except for in bitopertin (60 min pre-treatment time). The Black bars above the graphs indicated CBPT clusters of significant differences within subjects, i.e., between conditions. The Gray bars indicate clusters that have not reached significance threshold post-permutations. Data displayed as mean ± SEM

Next, we assessed how pharmacological agents that modulate distinct neurotransmitter systems impact ABRs in both wild-type (Fig. 2 and Additional file 1: Fig. S3) and Nrxn1α KO Sprague Dawley rats (Fig. 3 and Additional file 1: Fig. S4). In our first set of experiments, we used isoflurane anesthesia, as it is arguably the most-widely used choice for rodent ABR measurements. In order to investigate the effects of increasing GABAergic neurotransmission, we tested diazepam at 3 mg/kg (a γ2-containing GABA_A_ receptor enhancer; Fig. 2A and 3A), gaboxadol at 10 mg/kg (α4/6δ-containing GABA_A_ receptor agonist; Fig. 2B and 3B) and baclofen at 5 mg/kg (a GABA_B_ receptor agonist; Fig. 2C and 3C). To augment glycinergic neurotransmission we used bitopertin at 10 mg/kg (a GlyT-1 inhibitor; Fig. 2D and 3D). We used retigabine at 3 mg/kg (a pan-K_v_7 enhancer; Fig. 2E and 3E) to increase neuronal hyperpolarization and, therefore, to overall reduce synaptic outputs. Nicotine was used at 5 mg/kg (a nAChR agonist; Fig. 2F and 3F) in order to inhibit output of outer hair cells of the cochlea. Interestingly, we found that, compared to the vehicle control, none of the applied pharmacological agents clearly impacted ABRs in either wild-type or Nrxn1α KO Sprague Dawley rats. The only statistically significant effects were observed with nicotine on ABRs elicited at 90 dB and with retigabine on ABRs elicited at 80 dB. nicotine showed a modulation of the very late components of the ABRs in both wild-type (Fig. 2F; time window 5.4–6.25 ms time window, d = − 1.27, p = 0.037) and Nrxn1α KO Sprague Dawley rats (Fig. 3F; time window 6.6–7.9 ms; d = 0.97, p = 0.009), while retigabine only affected ABRs of Nrxn1α KO Sprague Dawley rats (Additional file 1: Fig. S4C, time window 3.6–5.9 ms, d =− 0.84, p = 0.01).

### ABRs are largely resistant to pharmacological modulations under medetomidine anesthesia

**Fig. 4:**
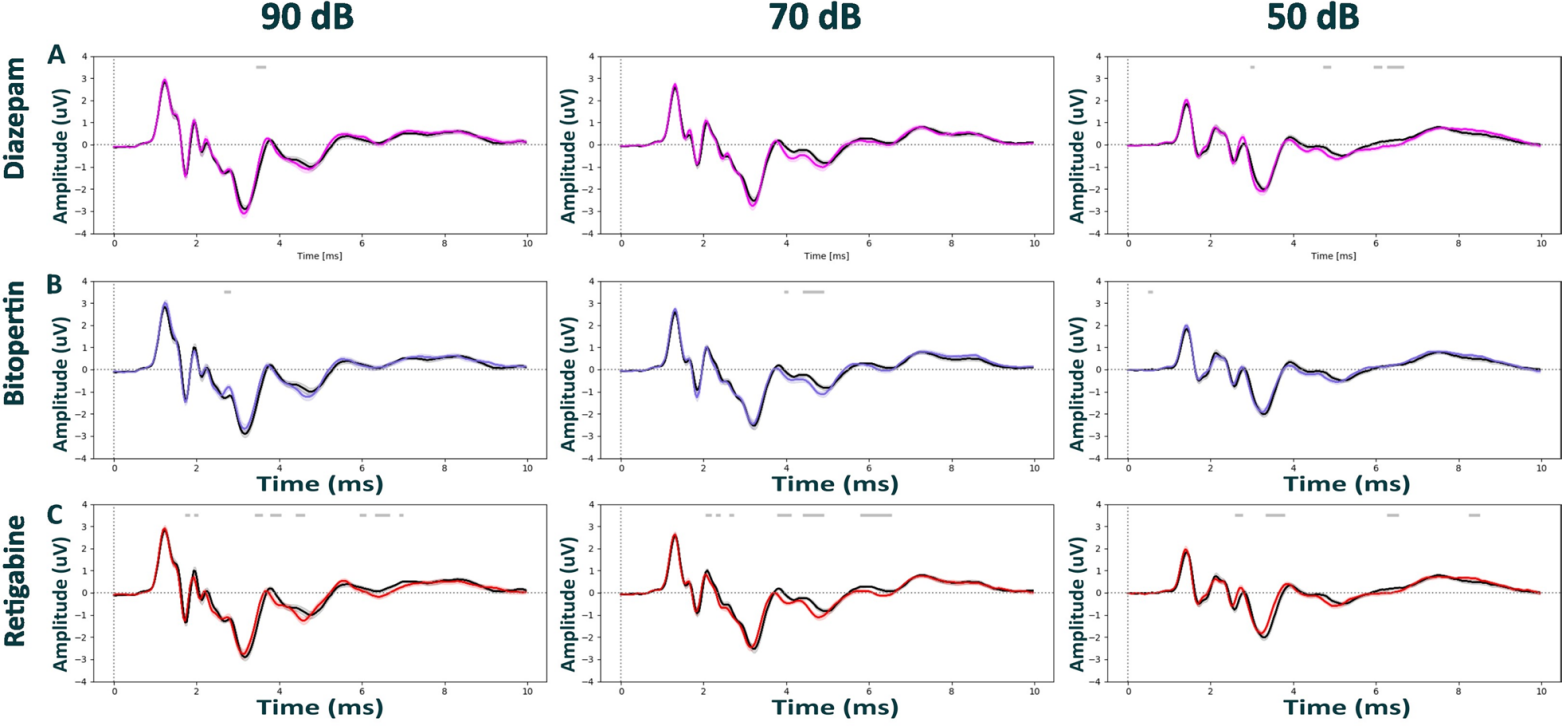
Auditory brainstem responses post pharmacological treatment in WT Sprague Dawley rats under medetomidine anesthesia. ABR waveforms across different stimulus intensities (90, 70, 50 dB) post intraperitoneal injection with diazepam (3 mg/kg) in magenta; (N = 12), bitopertin (10 mg/kg) in purple; (N = 12), retigabine (3 mg/kg) in red; (N = 12), or vehicle solution in black (0.9% saline + 0.3% Tween). Within each experimental block, dosing was counterbalanced, and applied 15 min prior to the ABR recordings for all compounds, except for in bitopertin (60 min pre-treatment time). Data displayed as mean ± SEM, was tested with unpaired CBPT. No robust significant differences were found between genotypes across anesthesia methods. Grey bars above the graphs indicate clusters of significant differences before CBPT-based correction for multiple comparisons, i.e., indicating statistical trends

**Fig. 5:**
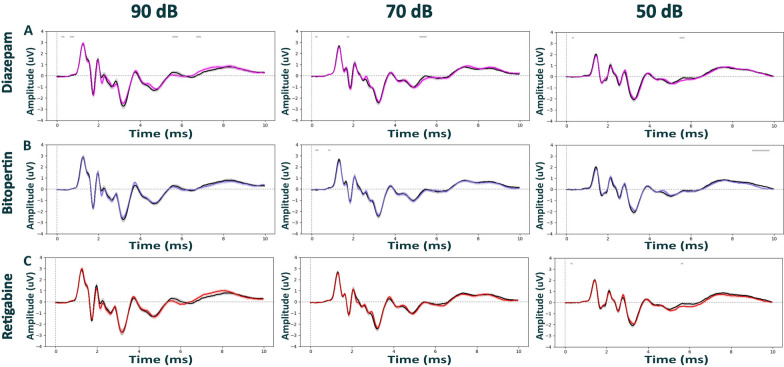
Auditory brainstem responses post pharmacological treatment in Nrxn1α KO Sprague Dawley rats under medetomidine anesthesia. ABR waveforms across different stimulus intensities (90, 70, 50 dB) post intraperitoneal injection with diazepam (3 mg/kg) in magenta; (N = 12), bitopertin (10 mg/kg) in purple; (N = 12), retigabine (3 mg/kg) in red; (N = 12), or vehicle solution in black (0.9% saline + 0.3% Tween). Within each experimental block, dosing was counterbalanced, and applied 15 min prior to the ABR recordings for all compounds, except for in bitopertin (60 min pre-treatment time). Data displayed as mean ± SEM, was tested with unpaired CBPT. No robust significant differences were found between genotypes across anesthesia methods. Grey bars above the graphs indicate clusters of significant differences before CBPT-based correction for multiple comparisons, i.e., indicating statistical trends

With the lack of pharmacological modulation observed under isoflurane, we next tested if ABRs could be modulated more clearly under medetomidine, a widely used anesthetic in functional imaging that is considered to preserve better network dynamics as compared to isoflurane or ketamine. To test this hypothesis, we focused on the three compounds diazepam (Fig. 4A and 5A), bitopertin (Fig. 4B and 5B) and retigabine (Fig. 4C and 5C). Like our observations under isoflurane, pharmacological modulation did not alter ABRs of both wild-type (Fig. 4 and Additional file 1: Fig. S5) and Nrxn1α KO Sprague Dawley rats (Fig. 5 and Additional file 1: Fig. S6) under medetomidine. The only statistically significant difference was found for retigabine in wild-type animals, reducing the amplitude of late components of ABRs elicited at 40 dB (Additional file 1: Fig. S5C; time window 3.8–5.5 ms, d = -1.44, p = 0.012; and time window 5.6–7 ms, d = -1.68, p = 0.013).

## Discussion

The current study explored the impact of different anesthetics and pharmacological tool compounds in wild-type and Nrxn1α KO Sprague Dawley rats and shows for the first time that rat ABRs are unaffected by diverse pharmacological modulators.

First, using three of the most widely used anesthetics for rodents, we confirmed that ABRs without additional pharmacological intervention are similar between adult Nrxn1α KO Sprague Dawley rats and their wild-type littermates. Our results align with our previous studies that probed ABRs in adult wild-type and Nrxn1α KO Sprague Dawley rats under isoflurane anesthesia only [33]. Our current study expands this finding by demonstrating the lack of genotypic differences also under ketamine/xylazine and medetomidine anesthesia. This finding is important, since previous studies showed that the choice of anesthesia (e.g., isoflurane vs. ketamine/xylazine) significantly affected ABR characteristics [41], raising the possibility that genotypic differences may be missed with just using one type of anesthesia with a specific mode of action. Isoflurane and ketamine/xylazine (the two most widely-used anesthetics for rodents ABRs [1]) share many molecular targets, including glycine receptors [42], GABA_A_ [42–45] and GABA_B_ receptors [46, 47], glutamate receptors [48–50] (including NMDA receptors [51–53]), and nACh receptors [54, 55]. All these receptors are widely expressed in the brainstem and along the auditory pathway [12, 13]. Any changes in these neurotransmitter systems may affect the transmission of auditory information from the cochlea to higher brain areas [54]. Indeed, Santarelli et al. showed that the latencies of ABR waves are significantly increased during isoflurane anesthesia compared to awake ABRs in Sprague Dawley rats [56]. These differences could be due to isoflurane reducing the glutamatergic neurotransmission at pre- and postsynaptic sites of inner hair cells [56] or by augmenting GABAergic inhibition within the auditory brainstem circuits. While similar circuit engagement can be expected with ketamine/xylazine, Ruebhausen et al. showed that isoflurane elevates hearing thresholds by around 30 dB more than ketamine/xylazine-based anesthesia [41]. This could be due to an additional effect of isoflurane by increasing blood flow to the brainstem and tissue perfusion [41, 57], in addition to a decrease in synaptic glutamate release [58], potentially reducing stimulus-driven activity [41]. As an alternative to isoflurane or ketamine/xylazine, we used medetomidine, an α2-adrenoceptor agonist, which is a common choice for fMRI studies as it preserved the dynamics of the brain better than α-chloralose or isoflurane [59, 60]. Indeed, previous studies show that medetomidine administration only marginally influences auditory-evoked potentials, picked up in the midbrain [61] and the cortex [62]. Other studies show that dexmedetomidine, a medetomidine isomer, demonstrated a minimal effect on ABRs in children [63] and it could be a better alternative for the commonly used oral chloral hydrate sedation [64].

A key point of the current study is that testing a diverse set of pharmacological modulators showed either none or only marginal effects on ABRs. This is surprising since the tool compounds, and doses used, engage receptors that are involved in signal transmission within auditory brainstem circuits. Only nicotine and retigabine treatment led to significant, but minor effects in the ABR. The effects of nicotine were confined to the very late phase of the ABR, resembling the activation of higher-order brain regions, and only at 90 dB stimulus intensity. While the major targets of nicotine (nACh receptors) are expressed at outer hair cells to regulate their sensitivity [65], no effects on the very early components of the ABR were evident. Therefore, our data imply for the action of nicotine on higher-order brain circuits to alter auditory processing [66]. For retigabine, we observed slightly reduced amplitudes of late components of the ABR at 80 dB, but not at 90 dB or at 70 dB. The volume-specific effect challenges the robustness and interpretability of the finding. More importantly, the fact that retigabine enhances voltage-gated potassium channels (such as K_v_7.4) expressed in the auditory brainstem [67], but does not clearly affect the ABR, highlights yet again the resistance of ABRs to pharmacological modulation. Our findings are in line with previous studies, showing a lack of ABRs and hearing threshold modulation with retigabine [39]. Beyond our findings with other compounds (such as diazepam, baclofen, or biopterin), the notion of a more general issue with pharmacological modulation of ABRs, is further supported by other rodent studies, demonstrating limited modulations of ABRs (slight increase in wave 1 amplitude, but no effects on latency) even with a high dose of opioids [68]. This is different from earlier studies demonstrating that theophylline [69] or cocaine [70] change ABR characteristics likely due to ototoxic rather than neuromodulatory effects.

An intuitive explanation for the lack of pharmacological modulation of ABRs in rodents is the “masking” effects of anesthesia, which may either block the target receptors and/or reduce neuronal dynamics to the extent that does not allow for further pharmacological modulation. We mitigated this caveat by using diverse anesthetic protocols, including medetomidine, which largely preserves network dynamics. Further support for the resistance of ABRs to pharmacological modulation comes from human and non-human primate studies which allow awake ABR experiments. In this context, Samra et al. showed in awake rhesus monkeys that neither Scopolamine nor Morphine intravenous injection could modulate the ABR waves [71]. In addition, studies in humans report no effects of anesthetic agents, or drugs such as benzodiazepines, propofol, and ketamine on ABRs [2, 72].Nonetheless, in our rodent study, a technical detail worth mentioning is the placement of the ground electrode under the contralateral ear in an open sound field configuration, there is a possibility that activation of the contralateral pathways interferes with the signals measured between the ipsilateral ear and the vertex. Given that key ABR features (e.g. 4–5 waves at defined latencies, and dependency of ABR amplitudes on stimulus intensity) are intact in our measurements and because the contralateral grounding introduces a systematic difference, we do not expect that a potential impact of genotype or pharmacological modulation on auditory brainstem processing would remain unnoticed in our measurements. Also, it is worth mentioning that our study was restricted to measuring ABRs with click sound stimulation protocols. Future studies could investigate whether tone ABRs at specific frequencies might be more sensitive to pharmacological modulation than click ABRs. Also, while dose selection rigorously followed the literature, higher doses could be explored in future work.

However, a more general concern with ABR measurements is that it primarily detects the neural response to sound onset and therefore might limit the identification of pharmacological effects, e.g. on later components of auditory signal processing. Therefore, complementary methods such as surface EEG recordings represent useful tools for studying the physiology of auditory signal processing.

Independent of these considerations, our study suggests that rodent ABR measurements are unsuited for testing auditory circuit modulation by diverse pharmacology. This conclusion is critical for drug development programs that aim to tackle auditory processing deficits, such as in psychiatric and neurodevelopmental disorders, where sensory abnormalities might stem from early-life disruption of auditory brainstem circuits [3].

### Supplementary Information

**Additional file 1: Fig. S1.** Comparison of auditory brainstem responses between Nrxn1α KO and wild-type littermates Sprague Dawley rats under isoflurane. **Fig. S2.** Comparison of auditory brainstem responses between Nrxn1α KO and wild-type littermates Sprague Dawley rats under ketamine/xylazine or medetomidine anesthesia. **Fig. S3. **Comparison of auditory brainstem responses between pharmacological modulations and vehicle in wild-type Sprague Dawley rats under isoflurane anesthesia. **Fig. S4. **Comparison of auditory brainstem responses between pharmacological modulations and vehicle in Nrxn1α KO Sprague Dawley rats under isoflurane anesthesia. **Fig. S5.** Comparison of auditory brainstem responses between pharmacological modulations and vehicle in wild-type Sprague Dawley rats under medetomidine anesthesia. **Fig. S6.** Comparison of auditory brainstem responses between pharmacological modulations and vehicle in Nrxn1α KO Sprague Dawley rats under medetomidine anesthesia.

## Data Availability

The datasets generated during and/or analyzed during the current study are available from the corresponding author on reasonable request.

## Abbreviations

ABRs: Auditory brainstem responses
Nrxn1α: Neurexin1α
AN: Auditory nerve
CN: Cochlear nucleus
SOC: Superior olivary complex
IC: Inferior colliculus
AC: Auditory cortex
LSO: Lateral superior olive
MNTB: Medial nucleus of the trapezoid body
CBPT: Cluster-based permutation tests
